# Art and Disease

**Published:** 1894-01-06

**Authors:** 


### Art and Disease.

### I.—POSSESSION OR HYSTERIA.

M. Charcot, by whose recent death the science of nervous complaints has sustained so great and irreparable a loss, has collected from ancient miniatures, tapestries, bas-reliefs, frescoes and engravings, many points of the deepest interest tending to show that the unhappy beings whose deliverance from evil spirits was one of the favourite subjects of mediæval painters were in fact victims to precisely the same symptoms as the hysteric patients under his daily observation in the hospital of La Salpêtrière.

In his work on "Demoniacs in Art" * he has furnished a description of some of the leading features of an attack of "la grande névrose." In its complete development such an attack presents four phases, and one or other of these the artist will be found commonly to select in depicting his demoniac. The first or epileptoid period, closely counterfeiting epilepsy, commences with tonic convulsions, loss of consciousness, momentary stoppage of the respiration, extreme pallor succeeded by flushes, swelling of the neck, convulsion of the eyeballs, distortion of the face, and often protrusion of the tongue, with foaming at the mouth. The body passes from extreme rigidity to convulsive movements extending to the muscles of the face, until the muscles relax, the breathing becomes stertorous, and the second period of wild movements, known as clownism, sets in. Almost every species of horrible contortion of the limbs and body may be noted in this phase of the disease. The physical force exerted is beyond all proportion to the normal powers, and this was formerly noted as a conspicuous sign of demoniac possession. The posture known as the "arc de cercle," in which the body is forcibly curved backwards until sometimes the head and heels alone rest on the bed, is one of the common contortions of this period, and the patient usually struggles to tear the hair and rend off all clothing. The third period is one of hallucination. The actions exhibited correspond to some idea present to the mind of the patient, joy, fear, horror, supplication, ecstasy are indicated by corresponding attitudes. The fourth period is one of gradual return to consciousness. The patient is subject to recurring delusions and moments of delirium, accompanied by convulsive movements. Violent cramp, producing loud cries of pain, is a frequent symptom at the close of an attack, readily suggesting to the bystander the idea of a demon torturing the possessed. In a regular attack these periods may succeed each other in the space of a quarter of an hour, but many variations in the order, vehemence, and duration of the phases are common. It is interesting to trace in the works of art of various centuries symptoms similar to those described, and attributed not only to demoniacal possession, but also in certain cases to divine inspiration.

In the most ancient representations of demoniac possession, however, the patient has nothing characteristic in either features or posture. The fact of his deliverance is indicated by the escape of the demon through his mouth or from the top of his head. One of the earliest of such representations is a carving in ivory, dating from the fifth century, on the cover of an Evangelium in the library at Ravenna. The demoniac is standing chained hand and foot, and the demon in the form of a little human figure, with uplifted hands, is half out of his head. The figure of the demon became an essential part of all such representations, and is seldom absent even in much later days. It takes the most varied forms, according to the caprice of the artist. Often it is dimly seen in a cloud of thick vapour escaping from the patient's mouth. Usually it is a conventional little devil, with horns, claws, wings, and tail, and a mocking countenance. Or the figure is covered with hair, or counterfeits some animal. In a fifteenth-century fresco, at Avignon, where St. Martial is represented in the act of exorcising a young man, the demon escapes in the form of a cat with bat's wings. In a book of engravings of 1615, St. Virgilius, Bishop of Salisbury, is shown expelling from a demoniac a monster without legs or arms, with a huge head, and butterflies' wings. When clothing is assigned to the demon, the orthodox livery is green.

But it is on the figures of the possessed themselves that the principal interest centres, and the criticism of one who combined the special knowledge of the physician with that of the art connoisseur is here specially valuable.

The earliest instance of a characteristic attitude assigned to a demoniac is found in a miniature of the eleventh century, preserved in the Cathedral of Aix-la-Chapelle. The young man is in the midst of a seizure; his body is violently curved backwards, his head and limbs convulsed. He is sustained by his father, and the attitude nearly approaches that noted above as common in hysterical attacks, and known as the "arc de cercle." Although attempts, more or less successful, can be traced in many of these earlier works of art to delineate actual traits which must have been observed in this class of sufferers, it is not till the time of the Renaissance that any really life-like representation was achieved. A miniature from a choir book (fifteenth century), preserved in the cathedral of Sienna, represents the casting forth of devils from several persons. One woman, awaiting her deliverance, is horribly contorted. The face is specially remarkable. The convulsion of the eye-balls with the pupils nearly hidden under the upper lid, is a very characteristic trait of a convulsive hysterical attack. The contracted brows and the furrows on the forehead express anguish; the nostrils are distended, and the teeth are visible in the widely-opened mouth. A fresco of Georgio, preserved at Sienna, shows the healing of a woman possessed by a demon at the shrine of a saint. The remarkable feature in this picture is the attitude of the woman's hand. She is bending backwards with upturned face; the right arm hangs down while the left is raised above her head, having the thumb and two first fingers outstretched, the other two fingers clenched into the palm. This attitude, proper to exorcism or benediction, can only be accounted for in the person of the demoniac herself on the theory of accurate observation of such sufferers by the artist. Amidst the wild movements of the convulsive crises of hysteria this pose of the fingers, M. Charcot notes, is frequently observed. "It is a gesture due solely to contraction, to the simultaneous action of all the tributary muscles of one of the great nerve trunks of the upper limb, the cubital nerve."

* "Les Démoniaques dans l'Art" par J. M. Charcot et Paul Richer.

